# Combination of Epinephrine with Esmolol Attenuates Post-Resuscitation Myocardial Dysfunction in a Porcine Model of Cardiac Arrest

**DOI:** 10.1371/journal.pone.0082677

**Published:** 2013-12-18

**Authors:** Qian Zhang, ChunSheng Li

**Affiliations:** Department of Emergency Medicine, Beijing Chao Yang Hospital, Capital Medical University, Beijing, China; Temple University, United States of America

## Abstract

**Background:**

Recent experimental and clinical studies have indicated that the β-adrenergic effect of epinephrine significantly increases the severity of post resuscitation myocardial dysfunction. The aim of the study was to investigate whether the short-acting β_1_-selective adrenergic blocking agent, esmolol, would attenuate post resuscitation myocardial dysfunction in a porcine model of cardiac arrest.

**Methods and Results:**

After 8 min of untreated ventricular fibrillation and 2 min of basic life support, 24 pigs were randomized to three groups (n = 8 per group), which received central venous injection of either epinephrine combined with esmolol (EE group), epinephrine (EP group), or saline (SA group). Hemodynamic status and blood samples were obtained at 0, 30, 60, 120, 240 and 360 min after return of spontaneous circulation (ROSC). Surviving pigs were euthanatized at 24 h after ROSC, and the hearts were removed for analysis by electron microscopy, Western blotting, quantitative real-time polymerase chain reaction, and terminal deoxynucleotidyl transferase–mediated dUTP nick end labeling (TUNEL) assay. Compared with the EP and SA groups, EE group had a better outcome in hemodynamic function, (improved dp/dt maxima and minima and cardiac output) (P<0.05), and improved oxygen metabolism (oxygen delivery and oxygen consumption) (P<0.05), which suggesting that EE can protect myocardial tissue from injury and improve post-resuscitation myocardial dysfunction. The protective effect of EE also correlated with reducing cardiomyocyte apoptosis, evidenced by reducing TUNEL-positive cells, increasing anti-apoptotic Bcl-2/Bax ratio and suppression of caspase-3 activity in myocardium.

**Conclusions:**

Esmolol, a short-acting β_1_-selective adrenergic blocking agent, given during CPR has significant effects on attenuating post resuscitation myocardial dysfunction. The current study provides a potential pharmacologic target for post resuscitation myocardial dysfunction.

## Introduction

Morbidity and mortality from cardiac arrest (CA) remains unacceptably high, yet effective treatments for CA have proven to be elusive [1]. Post-resuscitation myocardial dysfunction has been implicated as one of the major causes of fatal outcomes in patients who fail to survive hospitalization after initially successful cardiopulmonary resuscitation (CPR) [2], [3]. However, the mechanisms responsible for post-resuscitation myocardial dysfunction are not well-understood. Global myocardial ischemia during CA accounts for post-resuscitation myocardial dysfunction in rats, pigs, and human patients [4].

Epinephrine (EP) is a mixed adrenergic agonist, acting on α-adrenergic (α 1 and α 2) and β-adrenergic (β1 and β2) receptors. Evidence suggests that the important actions of EP for ROSC are mostly mediated by the α-adrenergic pathway, which increases coronary perfusion pressure via systemic arteriolar vasoconstriction, maintains peripheral vascular tone, and prevents arteriolar collapse [5]. In contrast to the α-adrenergic receptor effects, β-adrenergic receptor stimulation has been suggested to have a deleterious effect as stimulation of this pathway increases oxygen consumption, reduces sub-endocardial perfusion, and decreases post-resuscitation myocardial function [6]. Previous finding suggest that β-adrenergic antagonist may deserve consideration as a therapeutic intervention during advanced life support for prolonged ventricular fibrillation (VF) [7]. For the above mentioned reason, it was logical to assume that β-adrenergic blockade can reduce myocardial ischemic injury during CA and could result in higher resuscitation success. Esmolol is a β_1_-adrenergic receptor antagonist with a half-life of 9 minutes. In a recent study, esmolol led to smaller energy requirements for successful defibrillation, along with shorter resuscitation times and longer post-ROSC survival compared with EP [8]. It was therefore logical to assume that the co-administration of esmolol with EP during CPR would improve initial resuscitation success.

Increasing evidences demonstrate that apoptosis associates with the condition of ischemia/re-perfusion (IRI) and leads to myocardial dysfunction [9], [10]. Our previous study had also reported that the caspase-3 mediated apoptosis might be involved in the mechanism of post-resuscitation myocardial dysfunction [11]. Although the identities of the molecular signaling pathways that mediate ischemia-induced apoptosis are largely unknown, a common and critical event in the execution phase of apoptosis is the activation of the caspases [12], [13], which participate in a cascade where initiator caspases activate effectors caspases and ultimately cleave a set of proteins, causing disassembly of the cell. Activation of caspases maybe regulated directly or indirectly by Bcl-2 family proteins [14], [15]. Caspases and Bcl-2 family proteins have been proven to be involved in apoptotic cell death in cardiomyocytes [16].

The aim of the present study was to determine if administration of EP combined with esmolol during CPR will reduce the severity of global myocardial ischemic injury during the no-flow or low-flow state of CA and attenuate post-resuscitation myocardial dysfunction by reducing cardiomyocyte apoptosis in an established porcine model of CA. This work might provide insights into the development of a novel strategy to treat post-resuscitation myocardial dysfunction.

## Methods

### Animal preparation

This study was conducted with the approval of the Animal Care and Use Committee at the Chaoyang Hospital of Capital Medical University, China. Twenty-four inbred Wuzhishan miniature pigs (12–14 months of age, 30±2 kg) were used in this study. All animals were maintained in a specific pathogen-free environment in our facility, and were fed with standard chow and had free access to water. All animal experiments were performed in a humane manner, and also in accordance with the Institutional Animal Care Instructions. Pigs were randomized into three groups: esmolol group (esmolol 300 ug/kg + epinephrine 20 ug/kg per 20 mL dilution, bolus, EE group), epinephrine group (epinephrine 20 ug/kg, per 20 mL dilution, bolus, EP group), or saline group (20 mL dilution, bolus, SA group) (n = 8, per group) [17]. Animals were fasted overnight but were allowed free access to water. After premedication with 0.5 mg/kg intramuscular midazolam, anesthesia was induced by ear vein injection of propofol (1.0 mg/kg) and maintained in a surgical plane of anesthesia with intravenous infusion of pentobarbital (8 mg/kg per/hour). A cuffed 6.5-mm end tracheal tube was advanced into the trachea. Animals were ventilated with room air by a volume-controlled ventilator (Servo 900C; Siemens, Munich, Germany) at a tidal volume of 15 ml/kg and a frequency of 12 breaths/min. End-tidal PCO_2_ was measured by an inline infrared cacographic (CO2SMO plus monitor; Respirometric Inc, Murrysville, Pa). Respiratory frequency was adjusted to maintain end-tidal PCO_2_ between 35 and 40 mm Hg before inducing CA. Room temperature was adjusted to 26°C, and body temperature was maintained at 37°C under an infrared lamp. Aortic pressure was measured using a fluid-filled catheter that was advanced from the left femoral artery into the thoracic aorta. A Swan-Ganz catheter (7-Fr, Edwards Life Sciences, USA) was used to measure arterial pressure and was advanced from the left femoral vein and flow-directed into the pulmonary artery. Continuous cardiac output was measured with a cardiac output monitor (Vigilance II, Edwards Life Sciences). To induce VF, a 5-Fr pacing catheter was advanced from the right internal jugular vein into the right ventricle. Left ventricular function was measured using a fluid-filled polyurethane catheter that was introduced from the right carotid artery to the left ventricle for determine the maximum rate of left ventricular pressure increase (dp/dtmax) and the maximum rate of left ventricular pressure decline (−dp/dtmax) (BL-420F Data Acquisition & Analysis System, Chengdu TME Technology Co., Ltd.).

### Experimental protocol

After operation, the animals were allowed to equilibrate for 30 minutes. The temporary pacemaker conductor was inserted into the right ventricle through the right sheathing canal and connected to an electrical stimulator (GY-600A; Kaifeng Huanan Equipment Co, Ltd, Kaifeng, China) programmed in the S1S2 mode (300/200 ms), 40V, 8∶1 proportion, and 10-ms step length to provide a continuous electrical stimulus until VF [18]. Ventricular fibrillation was defined as an electrocardiogram showing waveforms corresponding to VF and a rapid decline in mean aortic pressure toward zero. After successful induction of VF, mechanical ventilation was discontinued. After 8 minutes of untreated VF, manual chest compressions were immediately initiated at a rate of 100 compressions per minute. CPR was performed by the same CPR technician from our laboratory, who compressed the porcine chest to approximately one-third of the anteroposterior diameter. The quality of chest compressions was controlled by a Heart Start MRx Monitor/Defibrillator with Q-CPR (Philips Medical Systems, Best, Holland) [19]. Eight minutes of VF was chosen because it is clinically relevant relative to emergency response system arrival and because by logistic regression models, this time period offers a realistic chance of influencing survival [20]. Ventilation was delivered by a bag respirator with room air, and the compression-to-ventilation ratio was 30∶2. After 2-minute CPR, the animals were randomly assigned to one of three groups (8 pigs per group), followed by a bolus injection of saline placebo (SA group),epinephrine combined with esmolol (300 ug/kg,per 20 mL dilution, EE group) or epinephrine (20 µg/kg, EP group) from central venous access. The study was blinded as to the medication used, and only the principal investigator, who did not take part in any resuscitation effort, knew the assignment of each animal. Furthermore, the investigators involved in data recording, data entry, and data analysis were also blinded to the allocation. If VF persisted after ten cycles of CPR (about 4 minutes), a 100 J (about 4 J/kg) shock (SMART Biphasic) was delivered. If the defibrillation attempt failed to attain ROSC, manual chest compressions were rapidly resumed for a further 2 minutes followed by a second defibrillation attempt. The second and subsequent shocks were delivered diphase 150 J. If spontaneous circulation was still not achieved, CPR was continued for a further 2 min, and defibrillation was attempted once more.

ROSC was defined as 10 consecutive minutes of maintenance of systolic blood pressure at 50 mm Hg. If spontaneous circulation was not restored within 30 min, we regarded the animal as dead [21]. All the animals received normal saline (10 mL/kg/h) intraoperatively to replenish fluid losses. After successful resuscitation, the animals were mechanically ventilated with 100% oxygen. With the exception of one jugular vein sheath that was used for fluid administration, all other vascular sheaths and end tracheal tubes were removed after a 6 h intensive care period. The animals were allowed to recover from anesthesia, and were then placed in observation cages and monitored for a further 18 hours. The animals were euthanatized with 10 mL of 10 mol/L potassium chloride intravenously following a bolus of 100 mg of propofol intravenously. Myocardial specimens were snap frozen in liquid nitrogen and stored at −80°C.

### Measurements

#### Hemodynamic Measurement

The hemodynamic parameters including heart rate (HR), cardiac output (CO), and mean aortic pressure (MAP), Left ventricular dp/dtmax and negative dp/dtmax were measured continuously, and we recorded the values at baseline, at 30 min, and at 1, 2, 4, 6 h after ROSC. Myocardial function was assessed from measurements of left ventricular pressure. Left ventricular dp/dtmax was measured and represents isovolemic contractility. Left ventricular −dp/dtmax was measured as an estimate of myocardial relaxation.

Arterial and mixed venous blood gas was examined (GEM Premier 3000 blood gas analyzer; Instrumentation Laboratory, Lexington, Mass); cardiac troponin I (cTnI) and lactate level analyses were drawn at baseline, at 30 min, and at 1,2,4 and 6 h after ROSC. Oxygen metabolism parameters, including oxygen delivery (DO_2_) and oxygen consumption (VO_2_), were calculated.

#### Measurement of serum lactate and cTnI concentrations

Blood obtained at baseline and after resuscitation were assayed for cTnI using a commercially available one step “sandwich” enzyme immunoassay method developed for human cTnI. Concentrations of serum lactate were measured by using lactate oxidize.

#### The neurological status evaluation

The neurological status of all surviving animals was evaluated at 24 hours after ROSC using the porcine cerebral performance categories (CPC) score. The investigators were blinded to the assessment of CPC. The CPC evaluation uses a 5-point scale to assess neurological function. CPC 1 indicates normal neurological function, with animals having no difficulty standing, walking, eating and drinking, and being alert and fully responsive to environmental stimuli; CPC 2 indicates mild neurological disability, with animals able to stand but exhibiting an unsteady gait, drinking but not eating normally, and responding more slowly to environmental stimuli; CPC 3 indicates severe neurological disability, with animals unable to stand or walk without assistance, not drinking or eating, and being awake but not responding normally to environmental stimuli; CPC 4 indicates coma; and CPC 5 indicates the brain death. CPC 1 and CPC 2 were considerable a favorable neurological outcome.

#### Harvest of the heart tissue

After the animals were euthanatized at 24 h after ROSC, heart was excised, the right ventricle and both atria were removed, and the left ventricle rapidly frozen by immersion in liquid N2 until required to measure the activities of Na^+^-K^+^-ATPase enzyme, Ca^2+^-ATPase enzyme, superoxide dis-mutase (SOD) and malondiadehyde (MDA).

#### Measurement of Na^+^-K^+^-ATPase and Ca^2+^-ATPase enzyme activity in myocardial tissue

Enzyme activity was assessed by measuring the optical density of Pi decomposed from ATP by the tissue protein according to the method of Isbir et al [22]. Na^+^-K^+^-ATPase and Ca^2+^-ATPase enzyme activities were determined using standard formulas.

#### Measurement of MDA and SOD in myocardial tissue

The individual tissue samples were added to 10 volumes of cold saline and were homogenized with a tissue homogenizer and centrifuged at 2,000 g for 15 min. MDA content was assayed to monitor the development of oxidative stress. A lipid per oxidation kit was used for determination of MDA. The assay is based on the reaction of a chromogenic reagent, N-methyl-2-phenylindole (R1), with MDA at 45°C. One molecule of MDA reacts with two molecules of reagent R1 to yield a stable chromophore with maximal absorbance at 586 nm. Values were given in nanomoles per milligram of protein. SOD enzyme activity was measured in homogenate of myocardial tissue. Enzyme activity was determined by the inhibition of the spontaneous oxidation of adrenaline to adrenochrome. Measurements were performed spectrophotometrically at 480 nm against sodium carbonate buffer (pH 10.2). The SOD enzyme activity values were expressed as national unit (NU) per milligram protein. The remaining tissue was preserved in 10% formaldehyde and 4% paraformaldehyde to observe pathologic changes in tissue ultra-microstructure under a transmission electron microscope (TEM).

#### Western blot analysis of Bcl-2, Bax, and caspase-3

A 100-mg frozen heart sample was homogenized in 2 mL of ice-cold buffer comprising 50 mM Tris HCl (pH 7.4), 150 mM NaCl,1% NP-40, 0.5% sodium deoxycholate,0.1% SDS, sodium orthovanadate, sodium fluoride, EDTA, leptin, and PMSF (final concentration is 1 mM). The tissues were homogenized and then centrifuged at 14,000 g for 15 min at 4°C. Nuclei and tissue were removed, and supernatant was separated and stored at −80°C until analysis. A total of 40 µg of protein was loaded onto 12% sodium dodecastyle sulfate-polyacrylamide gel electrophoresis gel in each sample. Western blotting was performed with the membranes blocked for 2 h with 5% non-fat milk and then incubated with the primary antibodies (diluted overnight at 4°C: Bax, 1∶500 (sc-70407; Santa Cruz Biotechnology, USA); Bcl-2, 1∶200 (MAB 4625; EMD Millipore, USA); caspase-3, 1∶500 (4051; Abcam Biotechnology, UK); and GAPDH, 1∶250 (Santa Cruz Biotechnology, USA). Blots were blocked and incubated at 4°C overnight with the specific primary antibody and for 1 h at room temperature with the appropriate horseradish peroxidase-conjugated secondary antibody. The immunoreactive bands were visualized on film and scanned. The data were analyzed by image software. Protein levels were normalized to β-actin and presented as ratio.

#### Quantitative real-time PCR assay for caspase-3

Total RNA was extracted from 50 to 100 mg of tissue according to the protocol described for the Bio Easy SYBR Green I Real-Time PCR Kit Manual (Bo Ri Technology Co., Ltd., China). Pre-incubation was performed at 95°C for 2 min, followed by amplification in 45 cycles at 95°C for 20 s, 59°C (caspase-3), 72°C for 30 s, and finally, during slow heating up, 72°C for 10 min. After the amplification, melting curve analysis with a temperature gradient from 65°C to 95°C was recorded every 0.5°C (hold for 5 s). The OD of the target genes was compared with that of GAPDH. The primer sequences of the expected PCR products were as follows: for caspase-3, sense 5-CATGGCCTGTCAGAAAATAC-3 and antisense 5-TAACCCGAGTAAAATGTGC-3; and for GAPDH, sense 5-GACCCAGAATACCAAGTGCAGATGTA-3, and antisense 5-CTGTTTCAGGAT-TTAAGGTTGGAGATT -3. Relative quantification is generally calculated with the 2-ΔΔCT formula by the comparative Ct method [23]. The mRNA expression of caspase-3 was determined by quantitative real-time PCR amplification of the cDNA sample. The absolute copy numbers of caspase-3 cDNA in the different groups were determined from the corresponding accurate standard curves.

#### TUNEL staining

TUNEL analysis was performed by an independent university histopathologic laboratory. The investigators were blinded to the intervention. TUNEL staining was carried out strictly according to the manufacturer's instructions (Roche Molecular Biochemicals) to identify the apoptotic cells in paraffin sections. For each specimen, cells with positive nuclei staining from five microscopic fields (400×) were counted. The total number of cardiomyocytes was also counted using light microscopy at a magnification of 400×. Quantitative analysis was presented as percentage of TUNEL-positive cardiomyocytes nuclei per total nuclei in each experimental group [24]. The results were evaluated separately by two different observers. The mean of the observations was considered to be the result.

#### Ultra structural analysis

The remaining tissue was preserved in 10% formaldehyde and 4% paraformaldehyde to observe pathologic and ultra structural changes of the myocardium under TEM (H-7650; Hitachi, Tokyo, Japan). The pathologic data were assessed by reviewers blinded to the experimental groups.

#### Statistical analyses

Results are expressed as the mean ±SD. Student *t* test was used for comparisons between every two groups. Differences at different time points within groups were compared with repeated-measures analysis of variance (ANOVA). In addition, the continuous variables were fixed to normal distribution and equal variances by Kolmogorov-Smirnov test and homogeneity of variance test. 6-hour survival, 24-hour survival and good neurological outcome between groups were performed a Chi-square analysis. A value of *P*<0.05 was considered as statistically significant. All analyses were conducted using the SPSS 17.0 software (SPSS Inc, Chicago III).

## Results

### Baseline status

Baseline hemodynamic measurements and oxygen metabolism measurements are shown in (Table 1). None of the variables (body weight, HR, MAP, CO, lactate concentration, DO_2_, VO_2_ and ERO_2_) differed significantly among the three groups (p>0.05).

**Table 1:** Baseline characteristics data among SA, EP and EE groups.

	SA group (n = 8)	EP group (n = 8)	EE group (n = 8)
Weight, kg	29.13±2.16	30.63±0.92	30.38±0.92
HR	99.00±7.44	101.38±8.30	100.50±10.04
MAP, mmHg	103.12±5.19	101.88±5.22	104.00±5.81
CO, L/min	2.86±0.22	2.99±0.20	2.99±0.19
DO_2_, ml/min	424±35	445±34	450±28
VO_2_, ml/min	112±12	112±9	115±12
ERO_2_, %	24.17±2.34	25.44±2.70	25.46±1.49
Lac, mmol/L	2.21±0.49	2.31±0.72	2.23±0.01

±SD.SA  =  saline, EP =  epinephrine, EE  =  epinephrine combined with esmolol. Values are mean

### Resuscitation Outcomes

Resuscitation outcomes are shown in Table 2. None of the 24 animals restored spontaneous circulation after initial defibrillation attempts. By comparison, the number of electric shock, defibrillation energy and time to ROSC were significantly lower in EP and EE group than in SA group (*p*<0.05), but there was no difference between EP and EE group (*p*>0.05). Six animals in the EP group and five animals in the EE group required dopamine support. 21 animals of three groups survived to 6 hours, and that 18 animals survived to 24 hours. There were no significant differences in 6-hour and 24-hour survival rate between the three groups. However,the increased incidence of the 24-hour survival rate with good neurological outcome in the EE group represents a promising trend. (a P value of 0.12 is obtained).

**Table 2:** Resuscitational outcome in SA, EP and EE groups.

	SA group (n = 8)	EP group (n = 8)	EE group (n = 8)
Number of shock	6.5±1.9	2.7±1.6^*^	2.6±1.3^*^
Energy of shock (J)	776±295.6	364.5±157.8^**^	351.4±139.9^**^
Time to ROSC (min)	10.0±3.8	6±2.2^**^	5±1.7^**^
6-hour survival	6	7	8
24-hour survival	5	6	7
Good neurologic outcome	3	2	6^*#^

±SD or number (n). SA  =  saline, EP =  epinephrine, EE  =  epinephrine combined with esmolol. ROSC  =  restoration of spontaneous circulation. *p<0.05, **p<0.01 vs.SA, ^#^P<0.05 vs. EP. (a Chi-square analysis has been utilized) Values are mean

### Left ventricular function evaluation by invasive hemodynamic studies and oxygen metabolism status

**Figure 1:**
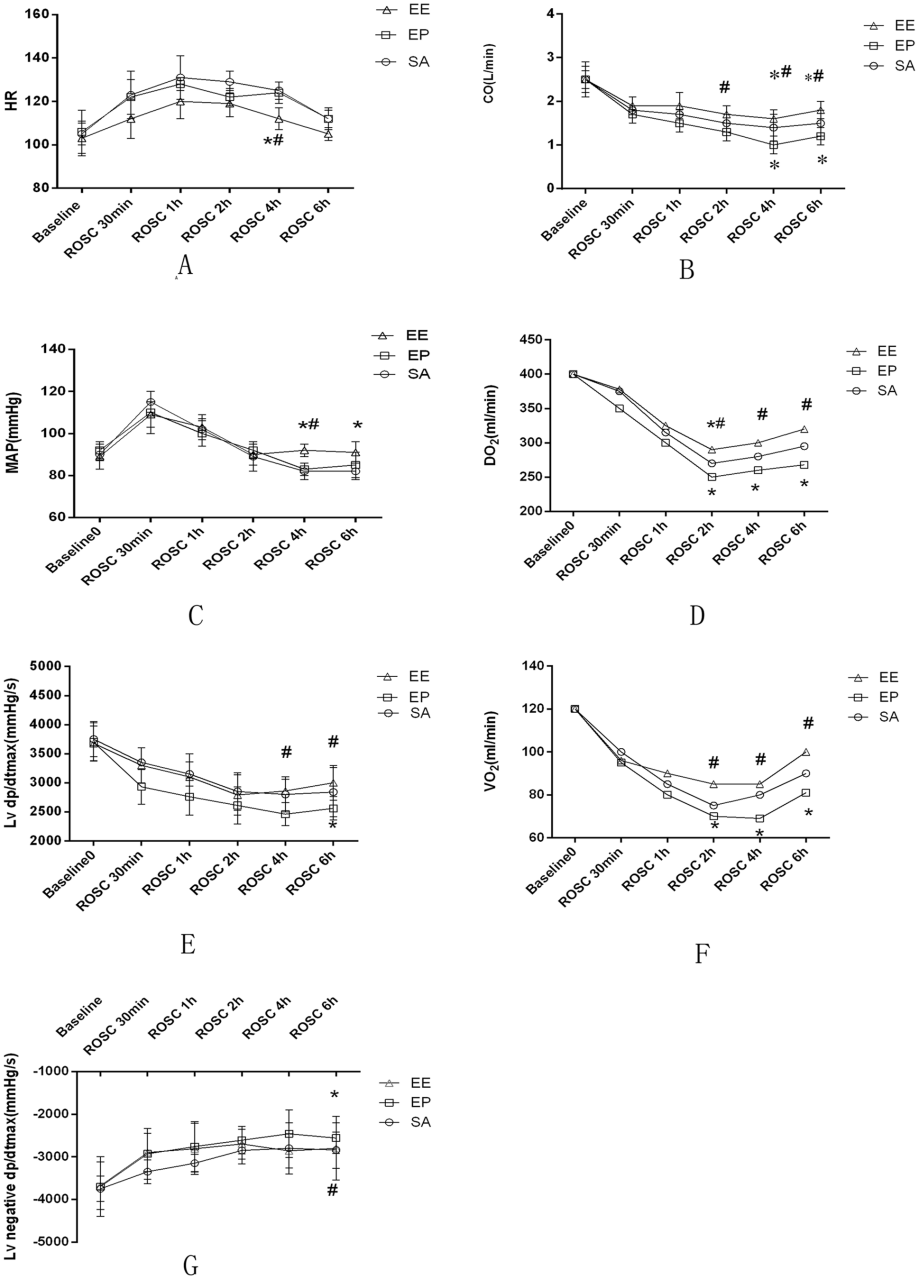
Evaluation of left ventricular function. (A) Heart rate (HR); (B)cardiac output (CO); (C)mean aortic pressure (MAP); (D) oxygen delivery (DO_2_); (E) Left ventricular dp/dtmax; (F) oxygen consumption (VO_2_); (G) Left negtive ventricular dp/dtmax; The values are reported as mean±SD. SA  =  saline, EP =  epinephrine, EE  =  epinephrine combined with esmolol. ROSC  =  restoration of spontaneous circulation. *p<0.05, **p<0.01 vs.SA, ^#^P<0.05 vs. EP (repeated-measures ANOVA).

After ROSC, HR was significantly lower in the EE group compared to the EP group and the SA group at 4 hours (*p*<0.05); however, there were no significant differences between the groups at any other time points (Fig1A). The values of CO was significantly higher in the EE group than in the SA group and the EP group at 4 h and 6 h (*p*<0.05); In addition, CO was significantly lower in EP group than in the SA group at 4 h and 6 h (*p*<0.05) (Fig1B). The values of MAP was significantly increased in the EE group compared to the EP group and SA group at 4 hours (*p*<0.05); however, there were no differences in MAP between the EE and EP groups at 30 min, 1 h and 2 h (Fig1C). Left ventricular dp/dtmax was significantly higher in the EE group than in the EP group at 4 h after ROSC. Left ventricular dp/dtmax and -dp/dtmax were significantly lower in the EP group than in the SA group and the EE group at 6 h after ROSC (Figure 1E, 1G). Oxygen metabolism measurements were shown among the three groups. DO_2_ and VO_2_ were significantly higher in the EE group than the EP group at 2, 4 and 6 h after ROSC (*p*<0.05) (Fig 1D, 1F).

### Serum cTNI and lactate concentrations

Serum cTnI and lactate concentrations were significantly increased throughout the study time points after ROSC compared with baseline values in all three groups (SA, EP, and EE groups) (*P*<0.05). However, the cTnI and lactate concentrations were lower in the EE group than in the EP group and the SA group at 2 h, 4 h and 6 h after ROSC (*P*<0.05, Table 3 and Table 4).

**Table 3:** Serum cTNI concentration (ng/mL) at baseline and throughout the study time points in SA, EP and EE groups.

Group	Baseline	ROSC0.5h	ROSC1h	ROSC2h	ROSC4h	ROSC6h
SA (n = 8)	0..03±0.02	0..55±0.22^Δ^	2.12±0.74^Δ^	3.99±1.24^Δ^	5.34±2.10^Δ^	8.71±3.20^Δ^
EP (n = 8)	0..03±0.02	0..49±0.18^Δ^	2.99±1.03^Δ^	4.12±1.06^Δ^	7.45±2.01^Δ^	9.41±2.41^Δ^
EE (n = 8)	0..03±0.01	0..32±0.19^Δ*#^	1.56±0.43^Δ*#^	2.12±0.74^Δ*#^	4.09±1.24^Δ*#^	5.33±2.32^Δ*#^

±SD.SA  =  saline, EP =  epinephrine, EE  =  epinephrine combined with esmolol. Δ p<0.05 vs. baseline, *p<0.05 vs.SA, #P<0.05 vs. EP (repeated-measures ANOVA). Values are mean

**Table 4:** Serum lactate concentration (mmol/L) at baseline and throughout the study time points in SA, EP and EE groups.

Group	Baseline	ROSC0.5h	ROSC1h	ROSC2h	ROSC4h	ROSC6h
SA(n = 8)	2.21±0.49	8.55±2.22^Δ^	6.96±1.32^Δ^	5.95±1.24^Δ^	4.53±1.45^Δ^	2.71±1.26^Δ^
EP(n = 8)	2.31±0.72	8.49±1.78^Δ^	7.73±2.02^Δ^	6.12±1.06^Δ^	5.63±2.02^Δ^	4.49±1.47^Δ^
EE(n = 8)	2.23±0.01	6.32±0.19^Δ*#^	5.73±1.42^Δ*#^	3.12±0.84^Δ*#^	2.76±0.62^Δ*#^	2.23±0.52^*#^

±SD. SA  =  saline, EP =  epinephrine, EE  =  epinephrine combined with esmolol. Δ *p*<0.05 vs. baseline, *p<0.05 vs.SA, ^#^P<0.05 vs. EP (repeated-measures ANOVA). Values are mean

### Left ventricle MDA, SOD, Na^+^-K^+^-ATPase, and Ca^2+^-ATPase content

The activities of left ventricle Na^+^-K^+^-ATPase, Ca^2+^-ATPase and the content of SOD were significantly increased in the EE group vs. the SA and EP groups at 24 h after ROSC. At the same time, the myocardial MDA content was significantly decreased in the EE group vs. the SA and EP groups (*P*<0.05; Table 5).

**Table 5:** Malondialdehyde content and activities of SOD, Na^+^-K^+^-ATPase and Ca^2+^-ATPase in left ventricular myocardium at 24 h after ROSC.

Group	SOD(NU/mg)	MDA(µmol/g)	Na^+^-K^+^-ATPase(U)	Ca^2+^-ATPase(U)
SA	11.58±2.57	19.32±4.17	4.97±1.04	3.65±1.01
EP	9.63±2.12	22.43±3.32	3.45±1.12	3.03±1.17
EE	21.27±3.67^*#^	14.30±2.53*^#^	6.89±1.37*^#^	4.58±1.43*^#^

±SD. SA  =  saline, EP =  epinephrine, EE  =  epinephrine combined with esmolol *p<0.05 vs.SA, ^#^P<0.05 vs. EP (repeated-measures ANOVA). Values are mean

### Ultra structural changes in cardiomyocytes

**Figure 2:**
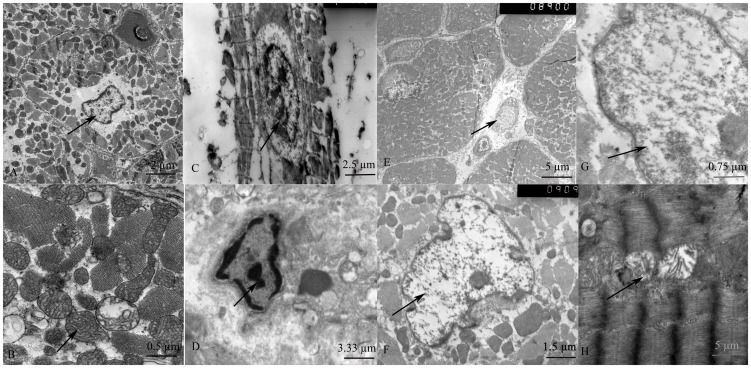
Ultra structure of the myocardium under an electron microscope. (Fig.2A, B): normal myocardial cell structure and normal mitochondria structure in the SHAM group (arrows); (Fig.2C, D): myocardial fiber and intercalated disk were obviously disordered, broken, even dissolved in the SA group (arrows); (Fig. 2E, F): myofibril organelles were extensively damaged and the myocardium exhibited progressive, severe deterioration in the EP group (arrows). (Fig. 2G, H): moderate edema occurred in the mitochondria and sarcoplasmic reticula in the EE groups (arrows).

Under TEM, normal mitochondria structures were displayed in the sham group (Fig 2A, B). The myocardial fiber and intercalated disk were obviously disordered, broken, even dissolved in the SA group 24 h after ROSC; most of the mitochondria were severely broken, even exhibiting vacuolar degeneration, cristae were vague, arranged irregularly, or disrupted. (Fig 2C, D). Myofibril organelles were extensively damaged and the myocardium exhibited progressive, severe deterioration in the EP group 24 h after ROSC (Fig 2E, F). Animals treated with EE exhibited little intracellular damage in the myocardium: partial nuclear chromatin condensation was observed, crest fracture reduced and moderate edema occurred in the mitochondria and sarcoplasmic reticula (Fig 2H, I).

### Effect of EE on protein level of Bcl-2, Bax, and caspase-3 activities of myocardial tissue

**Figure 3:**
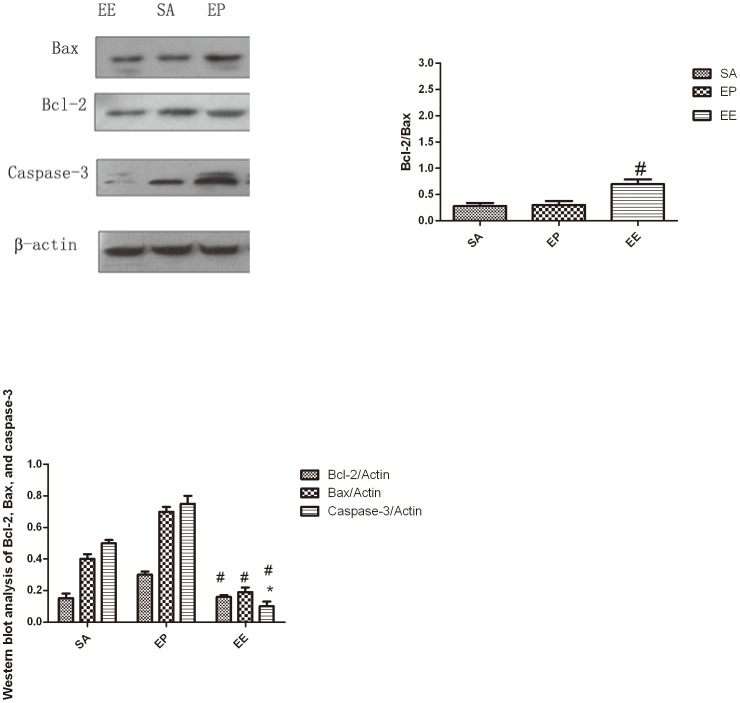
Effect of EE on protein level of Bcl-2, Bax, and caspase-3 activities. Western blots of (A) Expressions of Bax, Bcl-2 and caspase-3 proteins of myocardial tissue in the SA, EP and EE groups at 24 h after ROSC. (B) Expressions of Bcl-2/Bax proteins of myocardial tissue at 24 h after ROSC. (C) Quantification of Bax, Bcl-2 and active caspase-3 protein levels. The value represent mean ± SD. SA  =  saline, EP =  epinephrine, EE  =  epinephrine combined with esmolol. *p<0.05, **p<0.01 vs.SA, ^#^P<0.05 vs. EP (repeated-measures ANOVA).

Caspases activation, especially caspase-3 activation, has been reported in the programmed cell death (PCD) and in the pathological conditions such as IRI. Caspase-3 has been proved as the effectors' caspase of apoptosis. So we determined the caspase-3 activation in protein level by Western blot analysis. Figure 3 shows that the proteolytic activation of caspase-3 was significantly decreased in the EE group than in the EP group at 24 h after ROSC (*P*<0.05).

Bcl-2 family proteins play a critical role in the decision of the cell to die or survive by acting at multiple levels with a prompt impact on caspase activation [25], [26]. Therefore we also investigated the expression of the anti-apoptotic protein Bcl-2 in myocardium. In order to define additional mechanisms by which EE inhibits myocardium apoptosis, the amount of Bcl-2 family proteins expressed by myocardium was quantified by Western blot analysis. It was found that Bcl-2/Bax expression was decreased in myocardium and this decrease was significantly reversed by treatment with EE. Since decreased Bcl-2/Bax expression is associated with increased apoptosis, these results suggested that EE attenuate the apoptotic effects, in part, by up-regulation Bcl-2/Bax protein level than the EP group (*P*<0.05) (Fig 3).

### Caspase-3 mRNA expression

**Figure 4:**
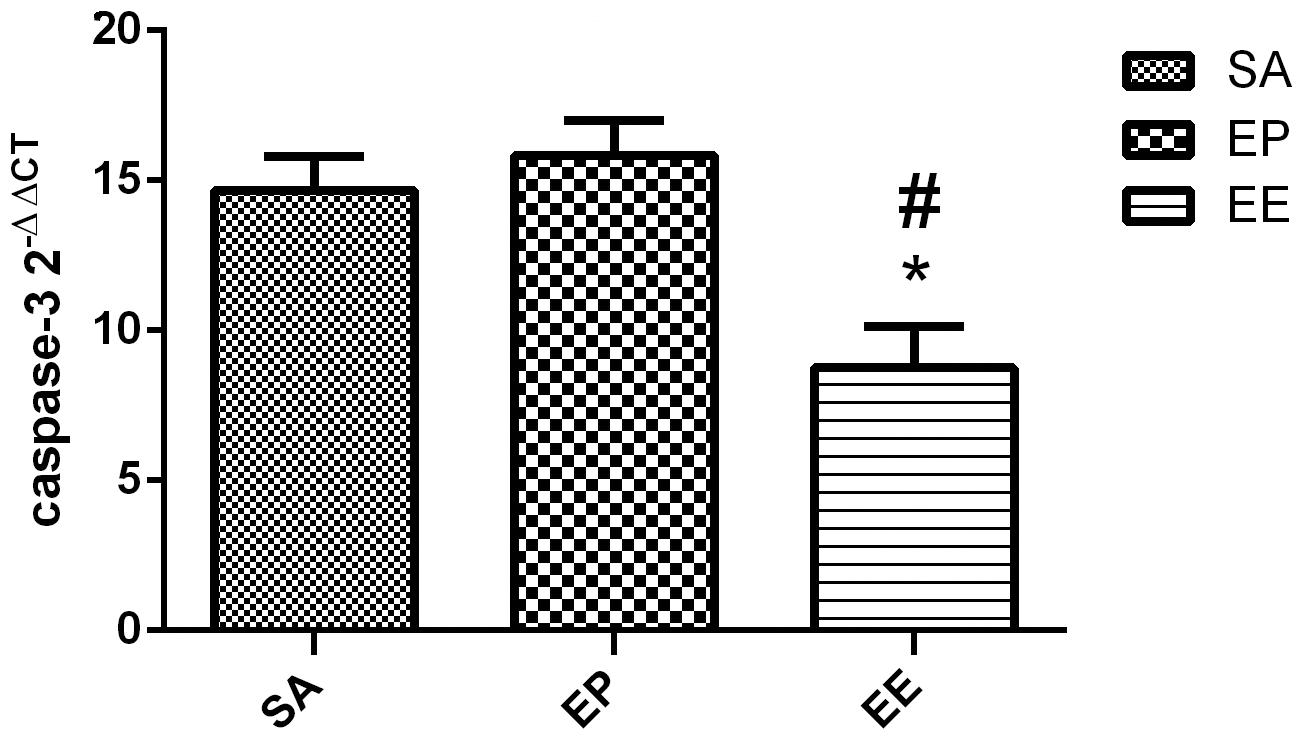
mRNA expressions of caspase-3. The mRNA expression of caspase-3 in the EE group was significantly reduced compared with the EP group. The value represent mean ± SD. SA  =  saline, EP =  epinephrine, EE  =  epinephrine combined with esmolol. *p<0.05 vs.SA, ^#^P<0.05 vs. EP (repeated-measures ANOVA).

To further investigate the potential mechanisms involved in apoptosis, RT-PCR analyses of caspase-3 mRNA expression in the each three group at 24 h after ROSC were conducted. Fig. 4 demonstrated that the significant down-regulation of caspase-3 mRNA expression induced by the EE group compared with the EP group (P<0.05) (Fig 4).

### TUNEL assay of cardiomyocyte apoptosis

**Figure 5:**
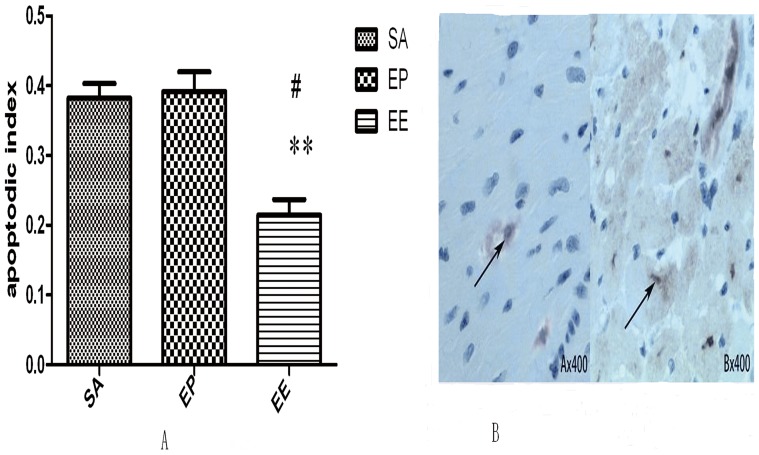
Changes in the number of TUNEL-positive myocytes in the experimental pigs at 24 h after ROSC. (A) Percentages of cardiomyocytes with TUNEL-positive nuclei. Cell apoptosis is another effect of I/R injury and can serve as a measure of the extent of I/R injury. In this study, we found that EE treatment alleviated myocyte apoptosis during I/R, evidenced by reducing amounts of TUNEL-positive cardiomyocytes, compared with SA group (*P*<0.01) and EP group (*P*<0.05). The value represent mean ± SD. SA  =  saline, EP =  epinephrine, EE  =  epinephrine combined with esmolol. *p<0.05, **p<0.01 vs.SA, ^#^P<0.05 vs. EP (repeated-measures ANOVA). (B) Representative TUNEL-stained sections. The brown nuclei indicate TUNEL-positive nuclei.

In the present study apoptotic cells were observed using TUNEL stain as a marker. TUNEL-positive cells in the EP, EE and SA groups were recognized in affected myocytic nuclei with chromatin condensation, and were observed in both core and marginalzones. These cells were more common in the EP group and the SA group, and were distributed across the lesion, whereas there were fewer TUNEL-positive cells in the EE group and the cells that were present were thinly scattered in the lesion. The brown nuclei in Figure 5 show TUNEL-positive staining the total cardiomyocytes per microscopic field. The results were evaluated separately by two different observers. The mean of the observations was considered to be the result (Fig 5).

## Discussion

The major findings of this study were as follows: (1) EE can attenuate post-resuscitation myocardial dysfunction through beneficial effects on anti-oxidation ability and energy metabolism. (2) The protective effect of EE correlated with a marked up-regulation of the anti-apoptotic protein Bcl-2/Bax and inhibited the activation of caspase-3 in myocardial tissue.

The severity of post-resuscitation myocardial dysfunction is minimized and survival is increased when systemic and coronary blood flows are promptly restored such that the duration and the severity of myocardial ischemia is minimized [27]. Killings Worth et al. had showed that esmolol administered immediately after defibrillation can improve the rate of ROSC and increase 4-hour survival after prolonged VF in pigs, because esmolol is a short-acting β_1_-blockade with a half-life of 9 min, whose period of action was similar to the duration of the catecholamine surge [28]. Also, Cammarata et al. administered esmolol in a rat model during CPR and concluded that initial cardiac resuscitation was improved, post-resuscitation myocardial dysfunction was minimized, and duration of post-resuscitation survival was increased [8]. Those results are in accordance with our findings, where 24-hour survival rate with good neurological function was expressed a promising trend in the EE group. Furthermore, the EE group had a better outcome in hemodynamic and oxygen metabolism parameters compared with the EP and SA groups, which suggesting that EE can protect myocardial tissue from IRI and improve post-resuscitation myocardial dysfunction.

CA represents the most severe shock state during which the delivery of oxygen and metabolic substrates is abruptly halted, and metabolites are no longer removed. And, CPR can be viewed as a process of whole-body IRI [29]. A large amount of oxygen free radicals (OFRs) produced during the perfusion of ischemic myocardium is the main cause for IRI in ischemic myocardium. The activity of SOD could reflect the in vivo scavenging capability of OFRs [30]. MDA is an end product of lipid peroxidation that causes cellular damage and disruption of cell membranes when tissue antioxidants are exhausted [31]. Normally, Na^+^-K^+^-ATPase is a major energy-using process that accounts for as much as 40% of the basal metabolism of the body [32]. Ca^2+^-ATPase enzyme is another sarcolemmal enzyme. Intracellular calcium loading is considered to represent the common denominator of IRI induced cell dysfunction and death [33]. Our present study found that the content of left ventricular myocardial SOD and the activities of Na^+^-K^+^-ATPase and Ca^2+^-ATPase were significantly increased in the EE group than in the SA group and the EP group at 24 h after ROSC. At the same time, the content of MDA was significantly decreased in the EE group than in the SA group and the EP group, which demonstrated that EE can attenuate post-resuscitation myocardial dysfunction through beneficial effects on anti-oxidation ability and energy metabolism.

There are three morphologically and biochemically distinct forms of cell death that occur in the heart: necrosis, apoptosis, and possibly autophagy. Autophagy is a cellular process that degrades long lived proteins and dysfunctional organelles. Necrosis is a passive form of cell death caused by ATP depletion and rapid disruption of cell membrane integrity resulting in spillage of intracellular contents into interstitial and extracellular space, which initiates inflamamation and induces damage to neighboring cells. In contrast, apoptosis is an energy requiring form of progammed cell death whereby damaged cells are removed without provoking inflamation. Apoptosis is characterdizen by chromatin condensation, DNA fragmentation, plasma membrane blebbing (i.e., externalization of phos-phatidylserine), and cell shrinkage due to reduction incytoplasm and organelles. Finally, membrane-bound apoptotic bodies containing cytosol and processed organ-ells are formed and then removed by macrophages viaphagocytosis [34]. Increasing evidences demonstrate that apoptosis is a critical cellular event involved in the pathogenesis of myocardial IRI [35], [36]. Bcl-2 (B cell lymphoma gene-2) family proteins play important roles in the regulation of apoptosis and are important modulators of cardiomyocytes apoptosis [37], [38]. The Bcl-2 family includes a growing number of proteins that serve as critical regulators of pathways involved in apoptosis, acting to either inhibit or promote PCD. It is the most characteristic member of the family that inhibits apoptosis. Constitutive expression of high levels of Bcl-2 protein enhances survival of many kinds' cells including cardiomyocytes on exposure to various adverse stimuli. Bax (Bcl-2-associated protein X) is the most characteristic death-promoting member of the Bcl-2 family. The Bax gene encodes a protein that is primarily localized to the cytosol and after apoptotic stimulation is translocated to the mitochondria. Homodimers of Bcl-2 or Bax associates with the mitochondrial membrane and affects membrane permeability [39].

A family of caspases is another key regulator of apoptotic signaling pathway. Caspases are the executioners in the process of apoptosis. They are cysteine proteases with specificity for aspartic acid and are divided in two subgroups: the upstream or initiator caspases and the downstream or effector caspases. Activation of the caspases proceeds by proteolytic cleavage of the expressed proform. The role of initiator caspases is to function as signaling molecules activating the effector caspases via proteolytic cleavages. The effector caspases are directly implicated in the execution of apoptotic cells. One of the downstream caspases is caspase-3. Once activated, it is thought to be one of these molecules that are responsible for the actual demolition of the cell during apoptosis [40]. Furthermore, down-regulation of Bcl-2/Bax expression might result in the activation of the caspase family of proteases, such as caspase-3, which is responsible for the induction of apoptotic cell death, leading to internucleosomal DNA fragmentation [41]. Therefore, Bcl-2/Bax and caspase-3 protein levels were examined by Western blot analysis to determine whether the regulators of apoptosis were involved in the mechanism of cardiomyocytes death induced by IRI (Fig 3). Since apoptosis represents an active, gene-directed mechanism, it should be possible to control this process for therapeutic purposes. The results of the present study suggest that EE decrease myocardial apoptosis by down-regulation of caspase-3 mRNA expression compared with the EP and SA groups. In addition, less apoptotic cardiomyocyte were observed under treatment with EE. These data indicate that EE can prevent IRI-induced cardiomyocyte apoptosis. All these findings further suggest a critical role of the EE in post-resuscitation myocardial dysfunction. It is of interest to investigate the detailed mechanisms by which EE regulating the apoptosis in the future.

## Conclusions

Based on these experimental studies, we conclude that (1) the administration of EE after prolonged VF improves the success of initial resuscitation, decreases myocardial injury and ameliorates myocardial ultra structure. (2) The protective effect of EE correlated with a marked up-regulation of the anti-apoptotic protein Bcl-2/Bax and inhibited the activation of caspase-3. (3) This study provides a novel treatment target for the protection of post-resuscitation myocardial dysfunction.

### Limitations of the study

In the interpretation of our findings, repetitive electrical shocks themselves may increase the severity of post-resuscitation myocardial dysfunction in settings of myocardial ischemia [42]. Because our studies were performed on an animal model in the absence of underlying cardiovascular disease, direct applicability to human patients cannot be ensured. Optimal doses and methods of administration of esmolol also deserve additional investigation. This information would be useful in applying the use of esmolol to CPR.
